# Supplementary material for: Multiscale socio-ecological networks in the age of information
Source: PLoS One. 2018 Nov 1;13(11):e0206672. doi: 10.1371/journal.pone.0206672 (PMC6211716; doi:10.1371/journal.pone.0206672)
Supplement: S1 File — This file includes one table (Table A: Summary statistics of the case study sites) and 9 figures (Fig A: Screenshot of the interactive web application; Fig B: Positions of the geolocated Flickr photographs; Fig C: Influence of the parameters in the identification of the Flickr user’s place of residence; Fig D: Final number of users per site; Fig E: Relationship between the distance and the normalized distance across sites; Fig F: Rankings of the case study sites according to their level of attractiveness; Fig G: Effect of the distance traveled on the socio-ecological interactions; Fig H: Effect of the spatial granularity on the metrics; Fig I: Effect of the spatial granularity on the spatial overlap between locals and visitors’ interactions. (PDF) [file pone.0206672.s001.pdf]

Supplementary information for Multiscale socio-ecological networks in the age of information

Maxime Lenormand[1], Sandra Luque[1], Johannes Langemeyer[2], Patrizia Tenerelli[1], Grazia Zulian[3], Inge Aalders[4], Serban Chivulescu[5], Pedro Clemente[6], Jan Dick[7], Jiska van Dijk[8], Michiel van Eupen[9], Relu C. Giuca[10], Leena Kopperoinen[11], Eszter Lellei-Kovács[12], Michael Leone[13], Juraj Lieskovský[14], Uta Schirpke[15], Alison C. Smith[16], Ulrike Tappeiner[17] and Helen Woods[7]

[1] Irstea, UMR TETIS, 500 rue JF Breton, FR-34093 Montpellier, France
[2] Institute of Environmental Science and Technology, Universitat Autnoma de Barcelona, C/ de les Columnes s/n, Campus UAB, 08193 Bellaterra, Spain
[3] European Commission, Joint Research Centre (JRC), Directorate D - Sustainable Resources, Unit D3 - Land Resources, Ispra, Italy
[4] The James Hutton Institute, Craigiebuckler, Aberdeen, AB15 8QH, UK
[5] National Institute for Research and Development and Forestry, Blvd. Eroilor 128, 077191, Voluntari, Ilfov, Romania
[6] Center for Environmental and Sustainability Research (CENSE), NOVA School of Science and Technology NOVA University Lisbon, Campus da Caparica, 2829-516, Caparica, Portugal
[7] Centre for Ecology & Hydrology, Bush Estate, Penicuik, EH26 0QB, UK
[8] Norwegian Institute for Nature Research (NINA), Høgskoleringen 9, 7034 Trondheim, Norway
[9] Wageningen University and Research, Environmental Research, P.O. Box 47, 6700 AA Wageningen, The Netherlands
[10] Research Center in Systems Ecology and Sustainability, University of Bucharest, Splaiul Independentei 91-95, 050095, Bucharest, Romania
[11] Finnish Environment Institute, P.O.Box 140, FI-00251 Helsinki, Finland
[12] Institute of Ecology and Botany, MTA Centre for Ecological Research, Alkotmány u. 2-4., 2163-Vácrátót, Hungary
[13] Research Institute for Nature and Forest (INBO), Havenlaan 88 bus 73, 1000 Brussels, Belgium
[14] Institute of Landscape Ecology, Slovak Academy of Sciences, Akademická 2, 949 01 Nitra, Slovakia
[15] Institute for Alpine Environment, Eurac Research, Viale Druso 1, 39100 Bolzano, Italy
[16] Environmental Change Institute, University of Oxford, Dyson Perrins Building, South Parks Road, Oxford OX1 3QY, UK
[17] Department of Ecology, University of Innsbruck, Sternwartestr. 15, 6020 Innsbruck, Austria

Photograph classification process

In order to ensure that only photographs representing an interaction between an individual and an ecosystem are considered, each photo has been manually validated and classified conforming to the Common International Classification of Ecosystem Services [1]. We relied on this typology to identify and classify socio-ecological interactions according to different types of cultural services that people benefits from ecosystems. For each case study, the interpretation of the photographs was performed by between 2 and 6 local experts. Photos that were not relevant included the following categories: a) wrong geographic location; b) people or pets as main subject in the foreground, not representing an outdoor activity; c) indoor, parking, private gardens; d) vehicles in the foreground; e) objects, signs and logos not related to the landscape; f) photo duplicate; g) bad photo where the subject cannot be identified. The photos were classified according to different categories: aesthetic enjoyment of landscapes (wide views of natural or different kind of environments), recreational activities (e.g. photographs of sport activities, such as skiing, hiking, climbing, camping), aesthetic enjoyment or existence of species (photographs of animals or plants), or intellectual experiences such as education, artistic inspiration or cultural heritage (e.g. photographs of scientific field work, traditional livestock feeding practices, lifestyle related to agricultural heritage).

More specifically, the subject of each photo was manually validated and classified according to the landscape identified in the picture. We used six landscape categories: agricultural and open landscape, sparse forest landscape, forested landscape, mountain landscape, anthropic infrastructures, water landscapes and wetlands. At the end of the process, $16,716$ photos taken by $2,967$ users between January 2000 and 2017 were classified. Note that 98% of the photos were taken after 2007.

Identification of the user's place of residence

To identify the place of residence of the $2,967$ Flickr users, we retrieved through the Flickr API information related to all the geo-located photos taken by these users worldwide. Then, we divided the world using a grid composed of 100×100 square kilometers cells in a cylindrical equal-area projection. We only considered photos with the most precise spatio-temporal Flickr accuracy level intersecting the grid and (Figure B). For each cell visited by a user we count the number of distinct months during which at least one photo was taken from this cell. The place of residence of a user is given by the cell in which the user was present the higher number of months. The identification of the user's place of residence process allows us to discard non reliable Flickr users (collective account or not regular Flickr user). A first coarse filter was applied to exclude collective accounts from the data by filtering out users traveling faster than a plane (750 km/h). Then, to ensure that a user shows enough regularity and that the assigned place of residence is the region of the world where he/she is really living, we applied two filters. We considered only users having more than $N = 6$ distinct months with at least one photo taken and a rate of presence at the place of residence higher than $\delta = 1/3$. Where δ is the ratio between the number of distinct months with at least one photo taken in the cell of residence and N. These values represents a good trade-off between being relatively sure about the users' residence area and keeping enough number of users to have proper statistics (Figure C). The algorithm used to extract most visited locations from individual spatio-temporal trajectories is detailed in [2] and the source code is available online[1]. The final number of users per site is displayed in Figure D.

[1] https://www.maximelenormand.com/Codes

Accessibility and attractiveness

For each user u, we compute the distance d_{us} between their place of residence, represented by the average position of all the photos taken from his/her cell of residence, and the centroid of the study site s he or she has visited represented by the average position of all photos taken in the site by all users. However, this distance between user's origin and visited site can be biased by the geography. Indeed, some case study sites are more isolated than others, implying differences in terms of accessibility among sites. To take this heterogeneity into account, we define a measure of accessibility λ_s as the average distance between the place of residence of every inhabitants on earth (estimated with the Global Human Settlement Population grid [3]) to the study sites. Hence, for every users u that have visited at least once the site s we can compute a normalized distance $\hat{d}_{us}$ taking into account the origin of u and the accessibility of s (Equation 1). All the distances have been computed with the Haversine formula based on longitude and latitude coordinates.

$$\hat{d}_{us} = \frac{d_{us}}{\lambda_s} \qquad (1)$$

The normalized distance is comprised between 0 and 3, $\hat{d}_{us} = 0.015$ corresponds roughly to a distance of 100 km (Figure S4 in Appendix). To ease interpretation the results are expressed in kilometers multiplying $\hat{d}_{us}$ by a factor 100/0.015. Some statistics about the study sites and their accessibility is presented Table A.

Table A: Summary statistics of the case study sites.

Site	Surface (km^2)	Accessibility (km)
Barcelona	7,822	7,092
Cairngorms	3,253	7,176
Carpathians	326	6,187
Costa Vicentina	895	7,701
Danube	5,782	6,083
De Cirkel	181	6,866
Dovre	2,271	6,830
French Alps	255	6,896
Kainuu	24,438	6,467
Kiskunsag	1,720	6,379
Loch Leven	97	7,169
Sierra Nevada	3,657	7,429
Stubai valley	265	6,662
Trnava	270	6,443
Vinschgau	491	6,688
Warwickshire	2,256	7,120

Interactive web application

An interactive web application has been designed to provide an easy-to-use interface to visualize socio-ecological interactions at different scales in the 16 case studies across Europe (Figure A). It was developed as part of a research project funded by the ALTER-Net network[1]. We focused on

[1] http://www.alter-net.info/

four aspects of the multiscale socio-ecological network: a representation of the spatial network at a world scale, a visualization of the spatial and temporal distribution of interactions per site, and, finally, a representation of the type of interactions (recreational activities and type of landscapes). The source code of the interactive web application can be downloaded from[3].

References

[1] R. Haines-Young and M. Potschin. Common international classification of ecosystem services (cices). *Nottingham: Report to the European Environmental Agency*, 2013.

[2] M. Lenormand, T. Louail, M. Barthelemy, and J. J. Ramasco. Is spatial information in ICT data reliable? *In proceedings of the 2016 Spatial Accuracy Conference, 9-17, Montpellier, France.*, 2016.

[3] European commission, joint research centre (jrc); columbia university, center for international earth science information network - ciesin (2015): Ghs population grid, derived from gpw4, multitemporal (1975, 1990, 2000, 2015). european commission, joint research centre (jrc) [dataset] pid: http://data.europa.eu/89h/jrc-ghsl-ghs_pop_gpw4_globe_r2015a.

Supplementary figures

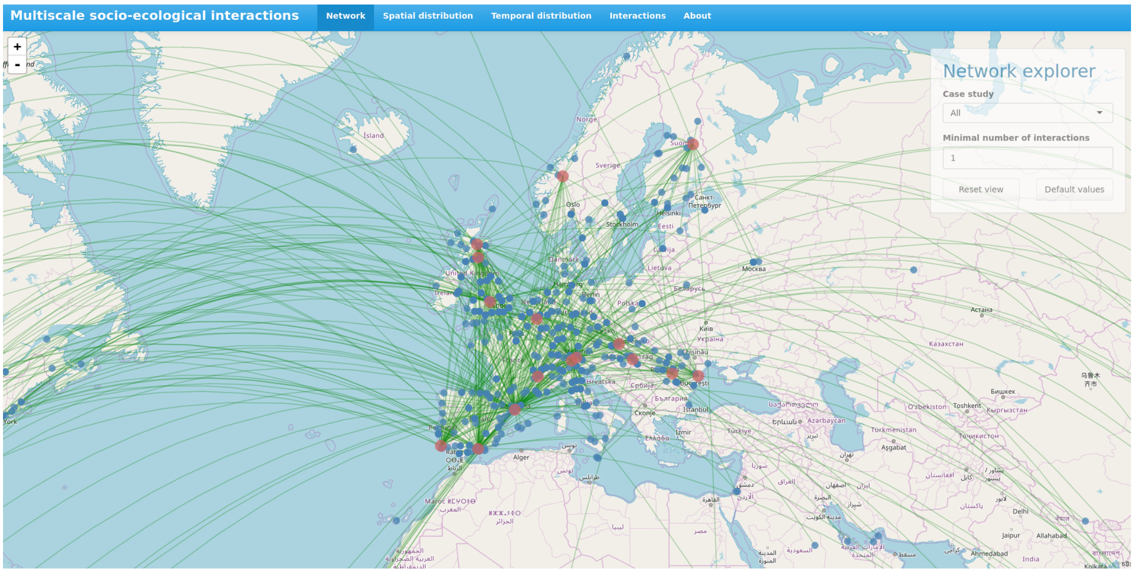

Figure A: **Screenshot of the interactive web application.**

[3] https://www.maximelenormand.com/Codes

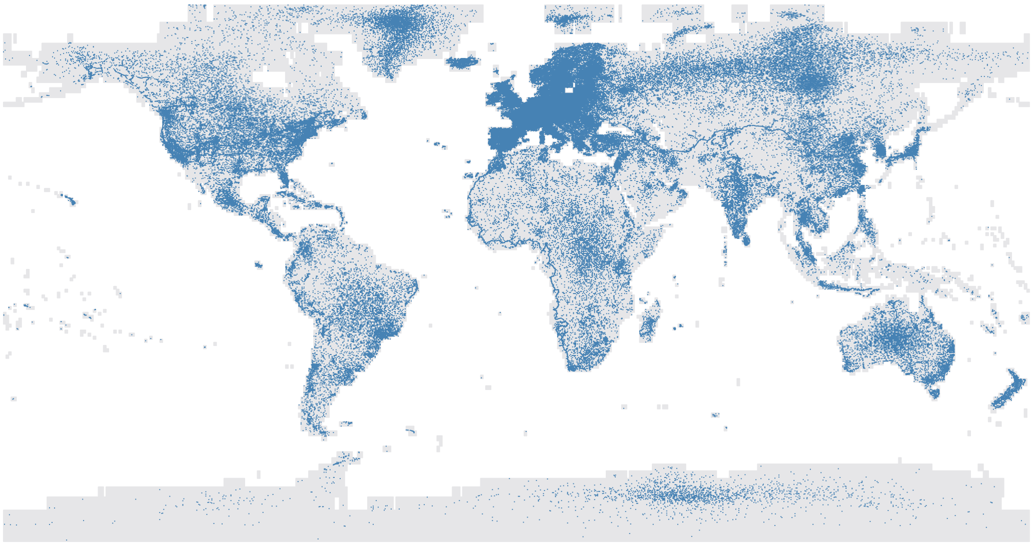

Figure B: **Positions of the geolocated Flickr photographs.** Each photo is represented as a point on the map location from which it was taken. Then, we divided the world using a grid composed of 100×100 square kilometers cells in a cylindrical equal-area projection. We only considered the $5,353,356$ photos intersecting the world grid composed of 100×100 square kilometers cells in a cylindrical equal-area projection (background).

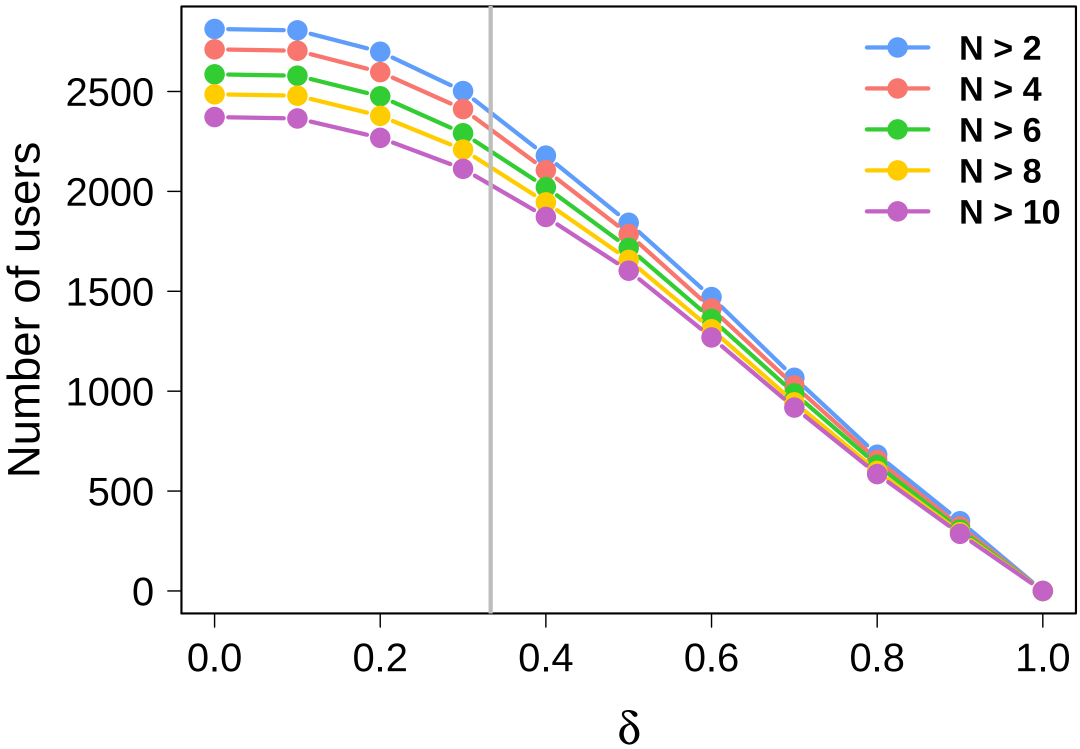

Figure C: **Influence of the parameters in the identification of the Flickr user's place of residence.** Number of reliable users as a function of δ for different values of N. The vertical bars indicate the value $\delta = 1/3$.

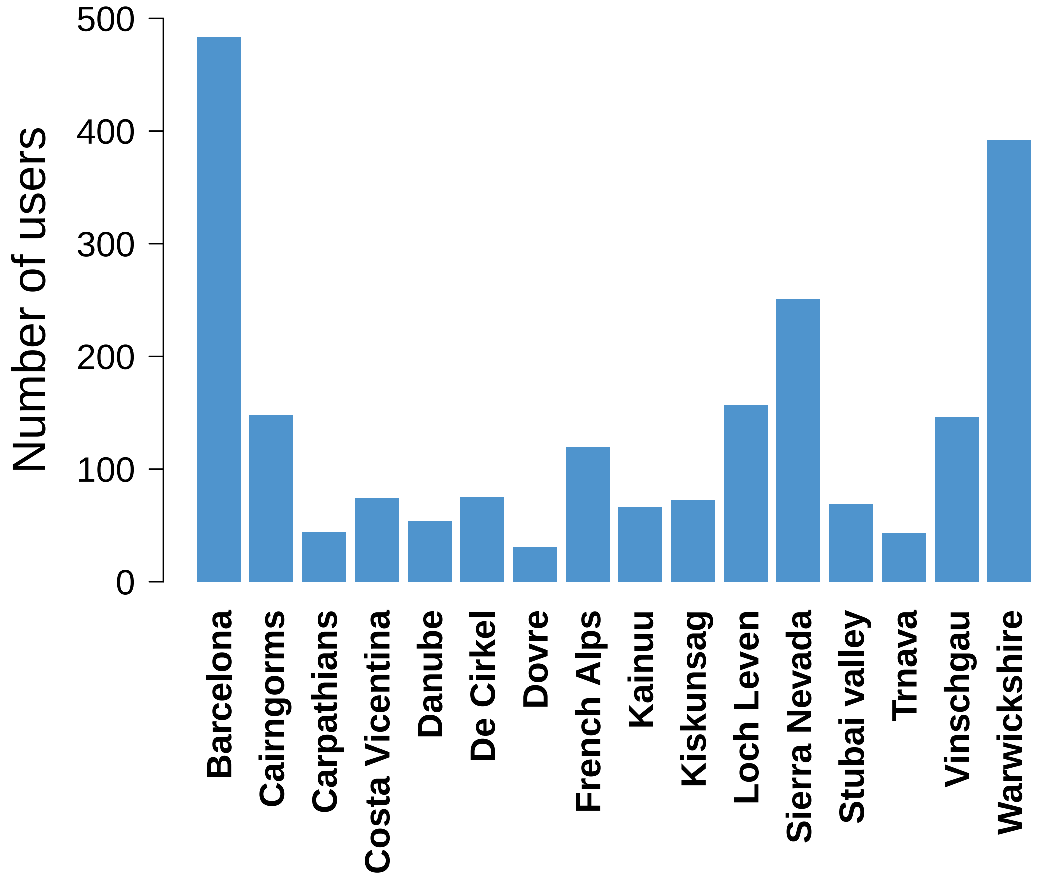

Figure D: **Final number of users per site.**

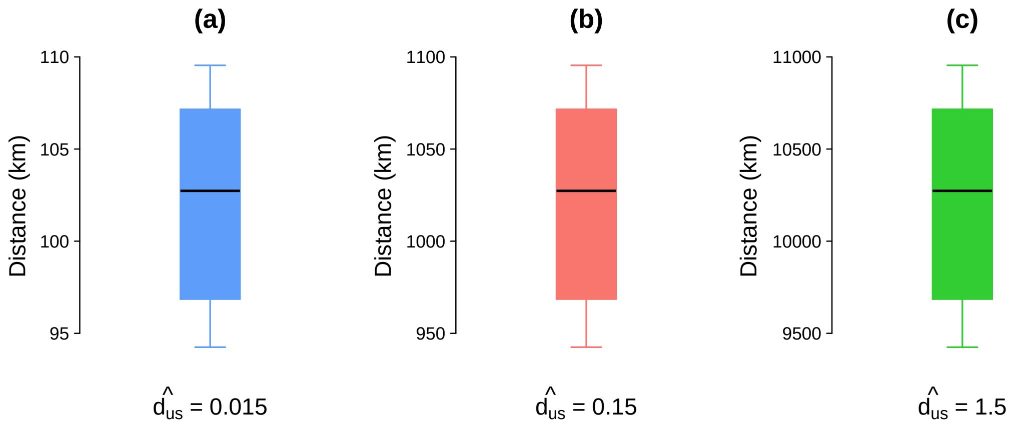

Figure E: **Relationship between the distance and the normalized distance across sites.** Boxplots of the distance from a site according to different normalized distance values $\hat{d}_{us}$ = 0.015 (a), $\hat{d}_{us}$ = 0.15 (b) and $\hat{d}_{us}$ = 1.5 (c). The boxplot is composed of the first decile, the lower hinge, the median, the upper hinge and the 9th decile.

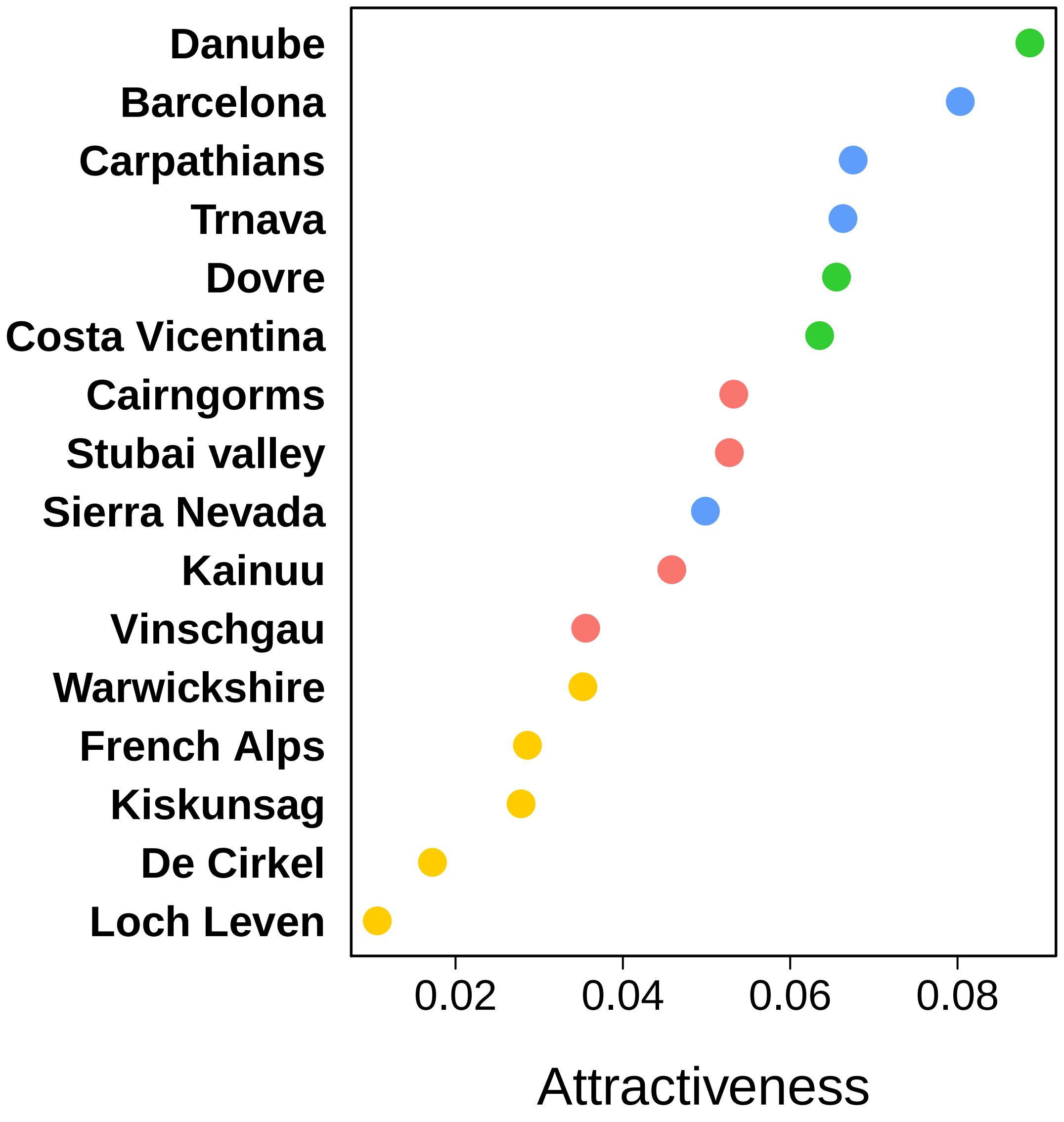

Figure F: **Rankings of the case study sites according to their level of attractiveness.** The attractiveness of a site is equal to the area above the CDFs and the colors corresponds to the cluster analysis presented in Figure 5.

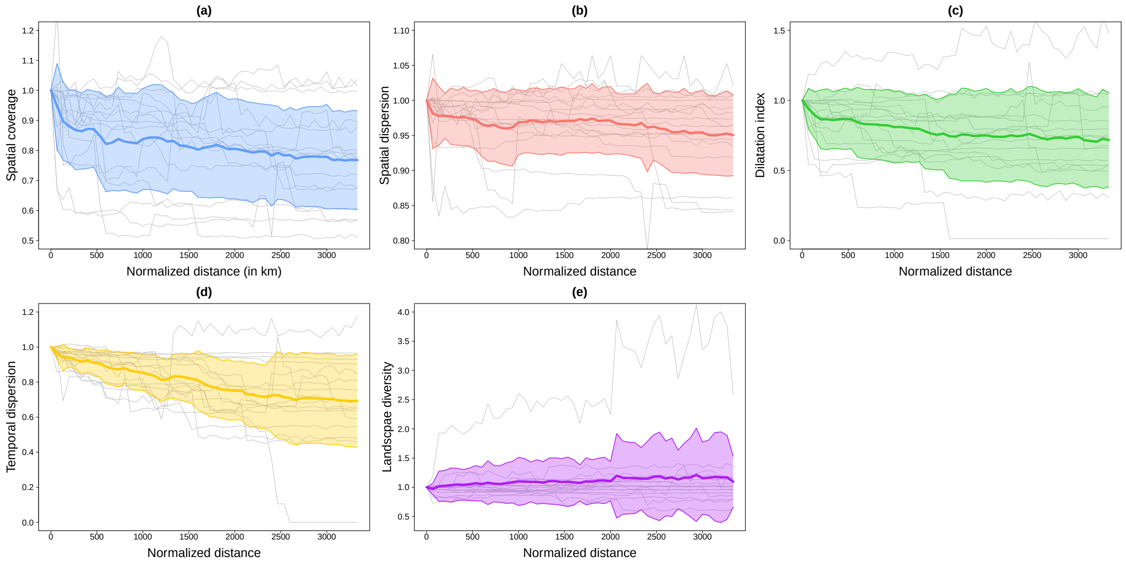

Figure G: **Effect of the distance traveled on the socio-ecological interactions.** Evolution of the spatial coverage (a), the spatial dispersion (b), the spatial dilatation index (c), the temporal dispersion (d) and the landscape diversity (e) as a function of the normalized distance. Each grey curve represents a case study. For each metric, the mean and standard deviation over the 16 case studies are displayed. All metrics are normalized by the value obtained with the null model.

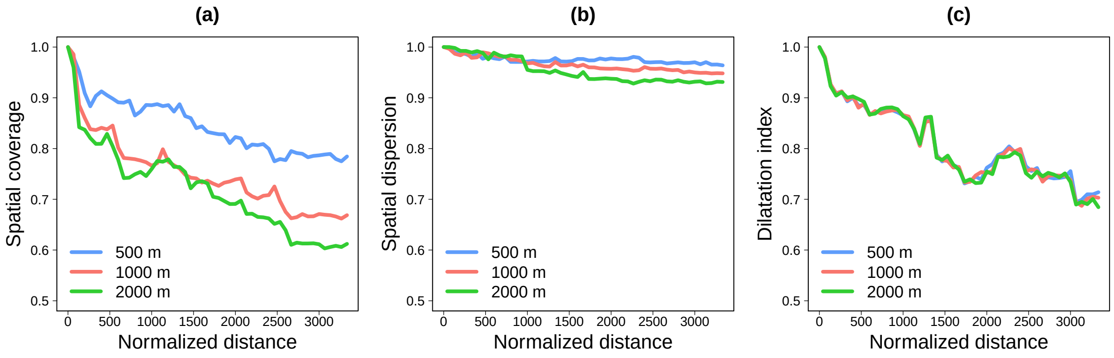

Figure H: **Effect of the spatial granularity on the metrics.** Evolution of the spatial coverage (a), the spatial dispersion (b), the spatial dilatation index (c) as a function of the normalized distance according to the cell size (500, 1000 and 2000 meters). For each metric, the median over the 16 case studies is displayed. All metrics are normalized by the value obtained with a random null model.

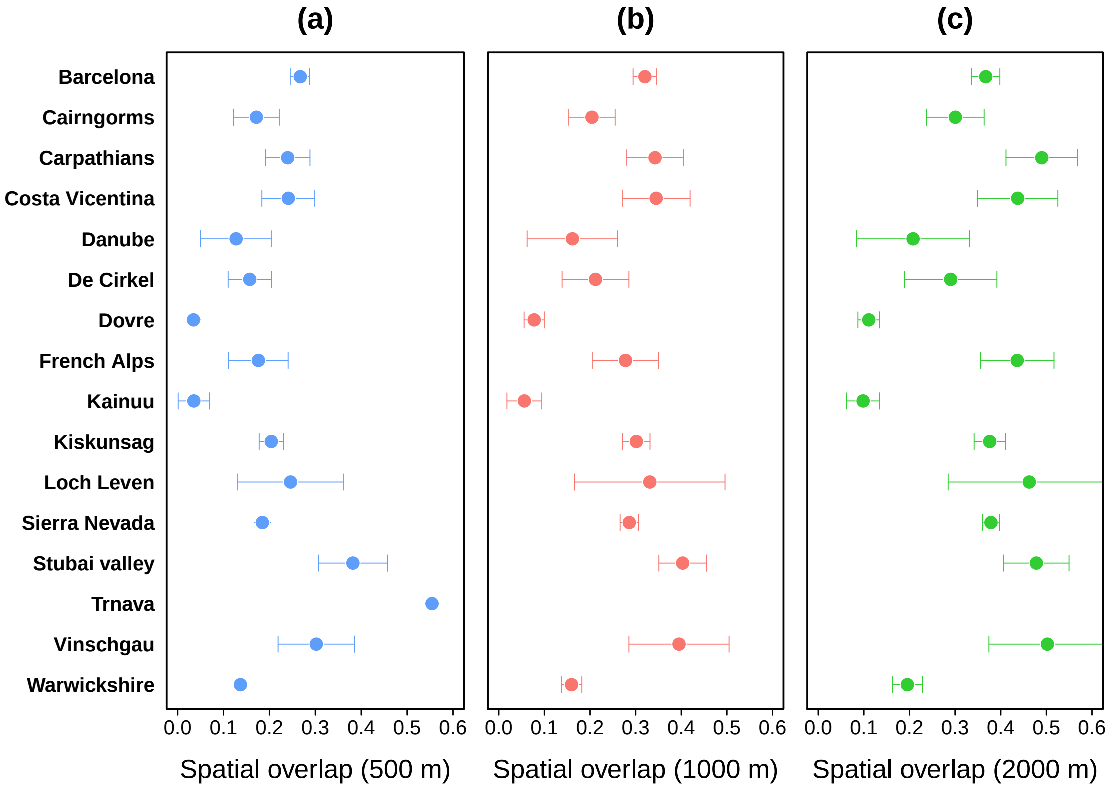

Figure I: **Effect of the spatial granularity on the spatial overlap between locals and visitors' interactions.** Different cell sizes are considered: (a) 500 meters; (b) 1000 meters; (c) 2000 meters. Locals and visitors are identified according to the normalized distance. In order to assess the impact of the threshold on the results we averaged the metrics obtained with threshold values ranging between 0.015 and 0.15. The error bars represent one standard deviation.
